# Crystal structures of di­iodido­bis­[(1*S*,5*S*)-4-mesityl-1,2,8,8-tetra­methyl-2,4-di­aza­bicyclo­[3.2.1]octan-3-yl­idene-κ*C* ^3^]palladium(IV) and di­chlorido­[(1*S*,5*S*)-4-mesityl-1,2,8,8-tetra­methyl-2,4-di­aza­bicyclo[3.2.1]octan-3-yl­idene-κ*C* ^3^](tri­phenyl­phosphane-κ*P*)palladium(IV)

**DOI:** 10.1107/S2056989015013055

**Published:** 2015-07-11

**Authors:** Eduard Rais, Ulrich Flörke, René Wilhelm

**Affiliations:** a Department Chemie, Universität Paderborn, Warburgerstrasse 100, 33098 Paderborn, Germany

**Keywords:** crystal structure, coordination compound, palladium complexes, NHC ligands

## Abstract

The mol­ecular structures of the chiral title compounds, [Pd(C_19_H_28_N_2_)_2_I_2_], (I), and [Pd(C_19_H_28_N_2_)Cl_2_(C_18_H_15_P)], (II), show a distorted square-planar coordination around the Pd^II^ atoms with two halogenide (Hal) ligands each and two N-heterocyclic carbene (NHC) ligands in (I) or one NHC and one tri­phenyl­phosphane ligand in (II). The deviations of the Pd^II^ atoms from the *L*_2_Hal_2_ best plane (*L* = NHC or tri­phenyl­phosphane ligand) are 0.206 (1) Å for (I) and 0.052 (1) Å for (II). The crystal packings exhibit inter­molecular C—H⋯Hal hydrogen bonds.

## Abstract 2

The Pd^II^ atoms in two N-heterocyclic carbene(NHC)/halogenide complexes show distorted square-planar coordination environments. In one complex, two NHC ligands are present, and the second complex contains an auxiliary tri­phenyl­phosphane ligand.

## Chemical context   

Six-membered N-heterocyclic carbene (NHC) ligands differ from the extensively reported five-membered analogues in several aspects. As a result of the increased N—C—N angle, the *N*-substituents exhibit a larger proximity to the metal atom, which can be an advantage for the transfer of chirality from the ligand to the product during a catalytic reaction or for the reductive elimination during the catalytic cycle (Cavallo *et al.*, 2005 ▸). The increased σ-donor ability of six-membered NHC ligands in comparison with their five-membered analogues can be helpful for catalytic applications or for the discovery of new catalytic reactions (Dröge & Glorius, 2010 ▸). Furthermore, NHC–metal complexes are less sensitive to dissociation, oxygen or elevated temperature compared to similar phosphane–metal complexes (Nolan, 2006 ▸). Notably, (NHC)_2_Pd complexes are known for their synthetic and catalytic applications (Schneider *et al.*, 2006 ▸; Türkmen & Cetinkaya, 2006 ▸). Structures of related bis­carbene complexes are known from Dunsford & Cavell (2014 ▸), Mayr *et al.* (2004 ▸) and Poulten *et al.* (2014 ▸).

We report herein on the syntheses and crystal structures of two N-heterocyclic-carbene (NHC)–Pd complexes {the chiral carbene being [(1*S*,5S)-4-mesityl-1,2,8,8-tetra­methyl-2,4-di­aza­bicyclo­[3.2.1]octan-3-yl­idene]} with two NHC-ligands in Pd(C_19_H_28_N_2_)I_2_, (I), and one NHC ligand in Pd(C_19_H_28_N_2_)(C_18_H_15_P)Cl_2_, (II).

## Structural commentary   

**Figure 1:**
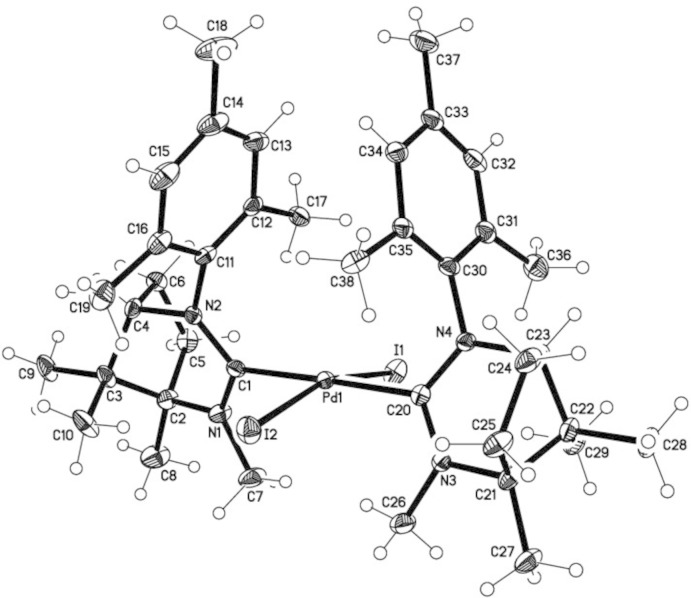
The mol­ecular structure of (I), with anisotropic displacement ellipsoids drawn at the 50% probability level.

The mol­ecular structures of the title compounds, (I) and (II), are shown in Figs. 1 ▸ and 2 ▸, respectively. The structure of (I) shows a distorted square-planar coordination environment around the Pd^II^ atom by the two N-heterocyclic carbene (NHC) and two iodido ligands. The deviation of the Pd^II^ atom from the I_2_C_2_ best plane is 0.206 (1) Å. The iodide ligands are *trans*-arranged and enclose an I—Pd—I angle of 163.275 (13) Å, whereas the C—Pd—C angle measures 178.32 (12)°. Pd—*X* bond lengths for *X* = C1, C20, I1, I2 are 2.070 (3), 2.079 (3), 2.6334 (4) and 2.6360 (4) Å, respectively. Other selected *X*—Pd—*X* angles are listed in Table 1 ▸. The mesityl ring planes make a dihedral angle of 32.7 (2)°.
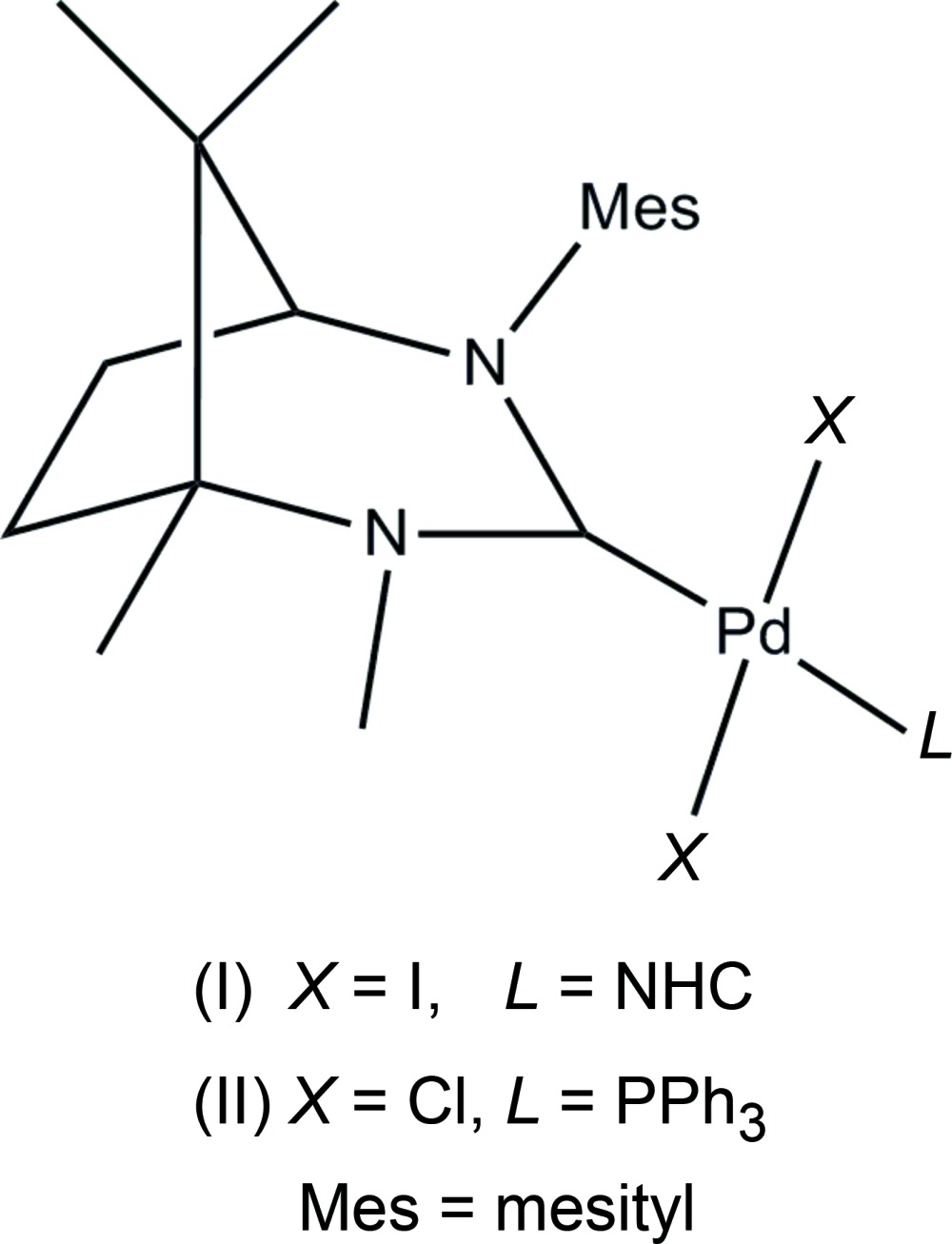


The structure of (II) also shows the Pd^II^ atom to be in a slightly distorted square-planar coordination by one NHC, one phosphine and two chlorido ligands. The deviation of Pd^II^ from the PCl_2_C best plane is only 0.052 (1) Å. The Cl ligands are also *trans*-arranged and enclose a Cl—Pd—Cl angle of 173.53 (9)° whereas the C—Pd—P angle measures 177.6 (2)°. Pd—*X* bond lengths for *X* = C1, P1, Cl1, Cl2 are 2.048 (7), 2.355 (2), 2.309 (2) and 2.311 (2) Å, respectively. Other selected *X*—Pd—*X* angles are listed in Table 2 ▸. The more pronounced deviation from planarity of the iodido complex is caused by the sterically more demanding iodido and the requirements of the mesityl-NHC ligands, respectively. In general, the NHC ligands in the structures of (I) and (II) exhibit no unexpected geometries.

## Supra­molecular features   

**Figure 3:**
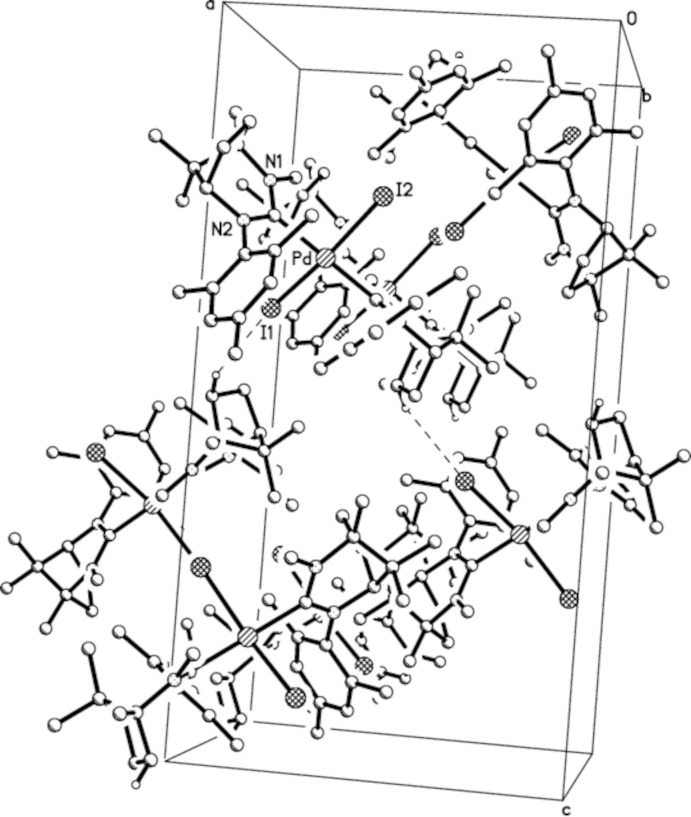
The crystal packing of (I), viewed approximately along [010], with inter­molecular hydrogen bonds shown as dashed lines. H atoms not involved in the hydrogen bonding have been omitted.

The crystal packing of (I) shows weak inter­molecular C5—H5*A*⋯I1 hydrogen bonds that link mol­ecules into zigzag chains extending parallel to [100] (Table 3 ▸ and Fig. 3 ▸).

**Figure 4:**
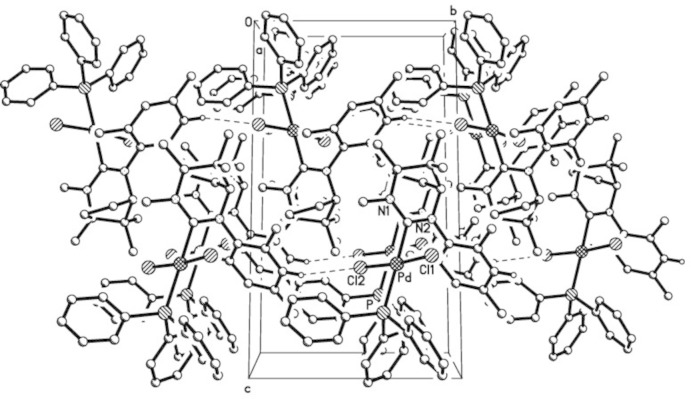
The crystal packing of (II), viewed along [100], with inter­molecular hydrogen bonds shown as dashed lines. H atoms not involved in the hydrogen bonding have been omitted.

In the crystal packing of (II), inter­molecular C15—H15*A*⋯Cl2 hydrogen bonds link mol­ecules into endless rows running parallel to [010]. Additionally, an intra­molecular C42—H42*A*⋯Cl2 bond is present (Table 4 ▸ and Fig. 4 ▸).

## Synthesis and crystallization   

The synthesis of the carbene precursor has been described by Koppenwallner *et al.* (2015 ▸). The title compounds (I) and (II) were prepared in similar ways using a stirred solution of (1*S*,5*S*)-4-mesityl-1,2,8,8-tetra­methyl-2,4-di­aza­bicyclo-[3.2.1]-oct-2-en-2-ium iodide (0.026 g, 0.063 mmol, 1 eq) and THF (3 ml) for (I) or 0.041 g, 0.099 mmol, 1 eq and 4 ml for (II) in a Schlenk tube. Potassium bis­(tri­methyl­sil­yl)amide dissolved in toluene (139 µl, 0.069 mmol, 1.1 eq, *c* = 0.5 mol/l) for (I) or 219 µl, 0.109 mmol, 1.1 eq, *c* = 0.5 mol/l for (II) was added and the mixture stirred for 1 h at room temperature under nitro­gen. After the remaining solid had been removed with a syringe filter, PdI_2_(PPh_3_)_2_ (0.050 g, 0.063 mmol, 1 eq) for (I) or PdCl_2_(PPh_3_)_2_ (0.070 g, 0.099 mmol, 1 eq) for (II) was added to the solution. The mixtures were stirred for 16–20 h under nitro­gen. Subsequently, the solvents were removed under vacuum and the residues washed three times with pentane (3 or 6 ml), dissolved in toluene and then carefully overlayed with pentane. Yellow crystals of Pd(NHC)_2_I_2_ (I) and colourless crystals of Pd(NHC)(PPh_3_)Cl_2_ (II) suitable for X-ray diffraction were obtained after several days.

## Refinement details   

Crystal data, data collection and structure refinement details are summarized in Table 5 ▸. Hydrogen atoms were clearly located from difference Fourier maps and then refined at idealized positions riding on the carbon atoms with *U*_iso_(H) = 1.2*U*(C_eq_) or 1.5*U*(C_eq_) (–CH_3_) and C—H 0.95–1.00 Å. All CH_3_ hydrogen atoms were allowed to rotate but not to tip.

## Supplementary Material

Crystal structure: contains datablock(s) I, II, global. DOI: 10.1107/S2056989015013055/wm5179sup1.cif

Structure factors: contains datablock(s) I. DOI: 10.1107/S2056989015013055/wm5179Isup2.hkl

Structure factors: contains datablock(s) II. DOI: 10.1107/S2056989015013055/wm5179IIsup3.hkl

CCDC references: 1411191, 1411190

Additional supporting information:  crystallographic information; 3D view; checkCIF report

## Figures and Tables

**Figure 2:**
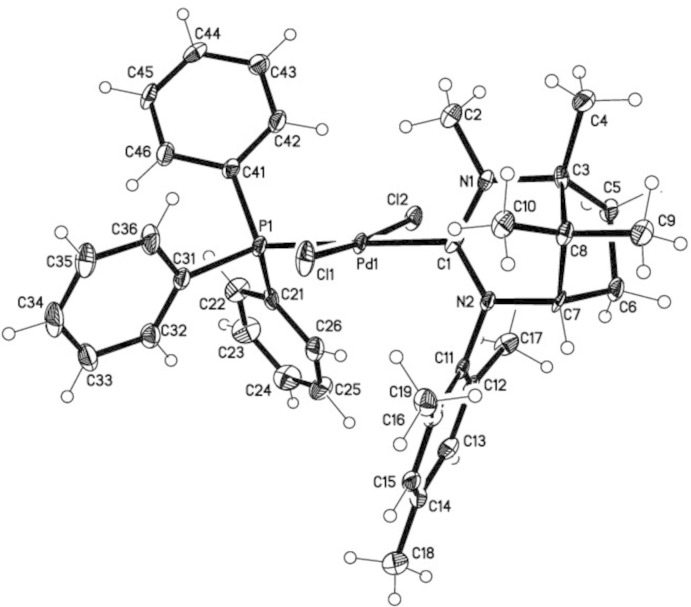
The mol­ecular structure of (II), with anisotropic displacement ellipsoids drawn at the 50% probability level.

**Table 1:** Selected bond angles (°) for (I)

C1—Pd1—I1	91.63 (9)	C1—Pd1—I2	89.05 (9)
C20—Pd1—I1	87.63 (8)	C20—Pd1—I2	91.23 (9)

**Table 2:** Selected bond angles (°) for (II)

C1—Pd1—Cl1	88.7 (2)	Cl1—Pd1—P1	93.58 (7)
C1—Pd1—Cl2	88.6 (2)	Cl2—Pd1—P1	89.18 (7)

**Table 3:** Hydrogen-bond geometry (Å, °) for (I)

*D*—H⋯*A*	*D*—H	H⋯*A*	*D*⋯*A*	*D*—H⋯*A*
C5—H5*A*⋯I1^i^	0.99	3.22	4.131 (5)	154

Symmetry code: (i) 
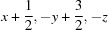
.

**Table 4:** Hydrogen-bond geometry (Å, °) for (II)

*D*—H⋯*A*	*D*—H	H⋯*A*	*D*⋯*A*	*D*—H⋯*A*
C42—H42*A*⋯Cl2	0.95	2.62	3.447 (8)	146
C15—H15*A*⋯Cl2^i^	0.95	2.71	3.535 (8)	146

Symmetry code: (i) 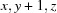.

**Table 5:** Experimental details

	(I)	(II)
Crystal data		
Chemical formula	[Pd(C_19_H_28_N_2_)_2_I_2_]	[Pd(C_19_H_28_N_2_)Cl_2_(C_18_H_15_P)]
*M*_r_	929.07	724.00
Crystal system, space group	Orthorhombic, *P*2_1_2_1_2_1_	Monoclinic, *P*2_1_
Temperature (K)	130	130
*a*, *b*, *c* (Å)	12.2480 (13), 13.3786 (14), 23.465 (2)	10.987 (3), 9.568 (2), 17.211 (4)
α, β, γ (°)	90, 90, 90	90, 107.478 (4), 90
*V* (Å^3^)	3845.0 (7)	1725.7 (7)
*Z*	4	2
Radiation type	Mo *K*α	Mo *K*α
μ (mm^−1^)	2.12	0.77
Crystal size (mm)	0.33 × 0.20 × 0.19	0.48 × 0.20 × 0.01

Data collection		
Diffractometer	Bruker SMART APEX	Bruker SMART APEX
Absorption correction	Multi-scan (*SADABS*; Bruker, 2002 ▸)	Multi-scan (*SADABS*; Bruker, 2002 ▸)
*T*_min_, *T*_max_	0.882, 0.985	0.710, 0.992
No. of measured, independent and observed [*I* > 2σ(*I*)] reflections	36757, 9177, 8551	13973, 8099, 6415
*R*_int_	0.047	0.089
(sin θ/λ)_max_ (Å^−1^)	0.658	0.658

Refinement		
*R*[*F* ^2^ > 2σ(*F* ^2^)], *wR*(*F* ^2^), *S*	0.029, 0.061, 1.01	0.053, 0.113, 0.92
No. of reflections	9177	8099
No. of parameters	414	393
No. of restraints	0	1
H-atom treatment	H-atom parameters constrained	H-atom parameters constrained
Δρ_max_, Δρ_min_ (e Å^−3^)	0.94, −0.33	1.00, −1.60
Absolute structure	Flack *x* determined using 3583 quotients [(*I* ^+^)−(*I* ^−^)]/[(*I* ^+^)+(*I* ^−^)] (Parsons & Flack, 2004 ▸)	Flack *x* determined using 2367 quotients [(*I* ^+^)−(*I* ^−^)]/[(*I* ^+^)+(*I* ^−^)] (Parsons & Flack, 2004 ▸)
Absolute structure parameter	−0.012 (10)	−0.02 (4)

Computer programs: *SMART* and *SAINT* (Bruker, 2002 ▸), *SHELXL2013* (Sheldrick, 2015 ▸), *SHELXTL* and *SHELXL* (Sheldrick, 2008 ▸) and local programs.

## supplementary crystallographic information

### (I) Diiodidobis[(1S,5S)-4-mesityl-1,2,8,8-tetramethyl-2,4-diazabicyclo[3.2.1]octan-3-ylidene-κC3]palladium(IV) Crystal data

**Table:**

[Pd(C_19_H_28_N_2_)_2_I_2_]	*D*_x_ = 1.605 Mg m^−^^3^
*M**_r_* = 929.07	Mo *K*α radiation, λ = 0.71073 Å
Orthorhombic, *P*2_1_2_1_2_1_	Cell parameters from 9243 reflections
*a* = 12.2480 (13) Å	θ = 2.2–25.0°
*b* = 13.3786 (14) Å	µ = 2.12 mm^−^^1^
*c* = 23.465 (2) Å	*T* = 130 K
*V* = 3845.0 (7) Å^3^	Prism, yellow
*Z* = 4	0.33 × 0.20 × 0.19 mm
*F*(000) = 1856	

### (I) Diiodidobis[(1S,5S)-4-mesityl-1,2,8,8-tetramethyl-2,4-diazabicyclo[3.2.1]octan-3-ylidene-κC3]palladium(IV) Data collection

**Table:**

Bruker SMART APEX diffractometer	9177 independent reflections
Radiation source: sealed tube	8551 reflections with *I* > 2σ(*I*)
Graphite monochromator	*R*_int_ = 0.047
φ and ω scans	θ_max_ = 27.9°, θ_min_ = 1.7°
Absorption correction: multi-scan (*SADABS*; Bruker, 2002)	*h* = −16→15
*T*_min_ = 0.882, *T*_max_ = 0.985	*k* = −17→17
36757 measured reflections	*l* = −30→30

### (I) Diiodidobis[(1S,5S)-4-mesityl-1,2,8,8-tetramethyl-2,4-diazabicyclo[3.2.1]octan-3-ylidene-κC3]palladium(IV) Refinement

**Table:**

Refinement on *F*^2^	Secondary atom site location: difference Fourier map
Least-squares matrix: full	Hydrogen site location: difference Fourier map
*R*[*F*^2^ > 2σ(*F*^2^)] = 0.029	H-atom parameters constrained
*wR*(*F*^2^) = 0.061	*w* = 1/[σ^2^(*F*_o_^2^) + (0.0269*P*)^2^] where *P* = (*F*_o_^2^ + 2*F*_c_^2^)/3
*S* = 1.01	(Δ/σ)_max_ = 0.002
9177 reflections	Δρ_max_ = 0.94 e Å^−^^3^
414 parameters	Δρ_min_ = −0.33 e Å^−^^3^
0 restraints	Absolute structure: Flack *x* determined using 3583 quotients [(*I*^+^)-(*I*^-^)]/[(*I*^+^)+(*I*^-^)] (Parsons & Flack, 2004)
Primary atom site location: structure-invariant direct methods	Absolute structure parameter: −0.012 (10)

### (I) Diiodidobis[(1S,5S)-4-mesityl-1,2,8,8-tetramethyl-2,4-diazabicyclo[3.2.1]octan-3-ylidene-κC3]palladium(IV) Special details

**Table:**

Geometry. All e.s.d.'s (except the e.s.d. in the dihedral angle between two l.s. planes) are estimated using the full covariance matrix. The cell e.s.d.'s are taken into account individually in the estimation of e.s.d.'s in distances, angles and torsion angles; correlations between e.s.d.'s in cell parameters are only used when they are defined by crystal symmetry. An approximate (isotropic) treatment of cell e.s.d.'s is used for estimating e.s.d.'s involving l.s. planes.
Refinement. Refinement of *F*^2^ against ALL reflections. The weighted *R*-factor *wR* and goodness of fit *S* are based on *F*^2^, conventional *R*-factors *R* are based on *F*, with *F* set to zero for negative *F*^2^. The threshold expression of *F*^2^ > σ(*F*^2^) is used only for calculating *R*-factors(gt) *etc*. and is not relevant to the choice of reflections for refinement. *R*-factors based on *F*^2^ are statistically about twice as large as those based on *F*, and *R*- factors based on ALL data will be even larger.

### (I) Diiodidobis[(1S,5S)-4-mesityl-1,2,8,8-tetramethyl-2,4-diazabicyclo[3.2.1]octan-3-ylidene-κC3]palladium(IV) Fractional atomic coordinates and isotropic or equivalent isotropic displacement parameters (Å^2^)

**Table:**

	*x*	*y*	*z*	*U*_iso_*/*U*_eq_	
Pd1	0.30214 (2)	0.548524 (17)	0.173876 (11)	0.01578 (6)	
I1	0.15512 (2)	0.628839 (18)	0.105990 (10)	0.02865 (6)	
I2	0.44142 (2)	0.517705 (19)	0.257697 (10)	0.02955 (6)	
N1	0.4408 (3)	0.7055 (2)	0.11875 (13)	0.0249 (7)	
N2	0.5017 (2)	0.54820 (19)	0.09716 (11)	0.0159 (5)	
N3	0.1463 (2)	0.5426 (2)	0.27152 (11)	0.0186 (6)	
N4	0.1155 (2)	0.4141 (2)	0.21027 (12)	0.0174 (6)	
C1	0.4287 (3)	0.6058 (2)	0.12539 (14)	0.0181 (7)	
C2	0.5186 (3)	0.7474 (3)	0.07584 (16)	0.0249 (8)	
C3	0.6274 (3)	0.6914 (3)	0.08531 (15)	0.0238 (8)	
C4	0.5915 (3)	0.5902 (2)	0.06160 (15)	0.0196 (7)	
H4A	0.6542	0.5427	0.0584	0.024*	
C5	0.4820 (3)	0.7145 (3)	0.01587 (16)	0.0253 (8)	
H5A	0.5003	0.7664	−0.0126	0.030*	
H5B	0.4023	0.7027	0.0150	0.030*	
C6	0.5446 (3)	0.6166 (2)	0.00311 (14)	0.0217 (7)	
H6A	0.4948	0.5637	−0.0109	0.026*	
H6B	0.6033	0.6275	−0.0252	0.026*	
C7	0.3834 (4)	0.7738 (3)	0.15652 (19)	0.0381 (11)	
H7A	0.3386	0.7355	0.1834	0.057*	
H7B	0.3364	0.8180	0.1340	0.057*	
H7C	0.4365	0.8141	0.1777	0.057*	
C8	0.5277 (4)	0.8621 (3)	0.0798 (2)	0.0418 (11)	
H8A	0.4558	0.8921	0.0733	0.063*	
H8B	0.5790	0.8863	0.0509	0.063*	
H8C	0.5540	0.8808	0.1178	0.063*	
C9	0.7216 (3)	0.7333 (3)	0.04938 (17)	0.0318 (9)	
H9A	0.7882	0.6952	0.0572	0.048*	
H9B	0.7334	0.8037	0.0592	0.048*	
H9C	0.7032	0.7278	0.0089	0.048*	
C10	0.6640 (4)	0.6908 (3)	0.14731 (16)	0.0353 (10)	
H10A	0.7332	0.6544	0.1506	0.053*	
H10B	0.6084	0.6579	0.1707	0.053*	
H10C	0.6741	0.7597	0.1605	0.053*	
C11	0.4947 (3)	0.4403 (2)	0.09457 (15)	0.0200 (7)	
C12	0.4196 (3)	0.3932 (2)	0.05859 (15)	0.0211 (7)	
C13	0.4284 (3)	0.2896 (3)	0.05110 (16)	0.0278 (8)	
H13A	0.3784	0.2570	0.0264	0.033*	
C14	0.5072 (4)	0.2329 (3)	0.07835 (19)	0.0335 (10)	
C15	0.5789 (4)	0.2819 (3)	0.11436 (18)	0.0345 (10)	
H15A	0.6321	0.2437	0.1342	0.041*	
C16	0.5761 (3)	0.3846 (3)	0.12272 (15)	0.0258 (8)	
C17	0.3302 (3)	0.4468 (3)	0.02708 (15)	0.0255 (8)	
H17A	0.2593	0.4283	0.0434	0.038*	
H17B	0.3322	0.4279	−0.0132	0.038*	
H17C	0.3407	0.5191	0.0306	0.038*	
C18	0.5154 (4)	0.1207 (3)	0.0700 (2)	0.0488 (13)	
H18A	0.4539	0.0976	0.0468	0.073*	
H18B	0.5134	0.0874	0.1072	0.073*	
H18C	0.5841	0.1047	0.0507	0.073*	
C19	0.6615 (3)	0.4310 (3)	0.16036 (17)	0.0330 (9)	
H19A	0.6937	0.3795	0.1848	0.049*	
H19B	0.6278	0.4828	0.1841	0.049*	
H19C	0.7186	0.4611	0.1367	0.049*	
C20	0.1737 (3)	0.4952 (2)	0.22313 (14)	0.0164 (7)	
C21	0.0656 (3)	0.4997 (2)	0.31324 (14)	0.0219 (7)	
C22	−0.0324 (3)	0.4653 (3)	0.27739 (15)	0.0217 (7)	
C23	0.0239 (3)	0.3771 (2)	0.24731 (15)	0.0217 (7)	
H23A	−0.0294	0.3359	0.2252	0.026*	
C24	0.0744 (3)	0.3173 (3)	0.29680 (16)	0.0280 (9)	
H24A	0.1359	0.2753	0.2836	0.034*	
H24B	0.0193	0.2743	0.3156	0.034*	
C25	0.1140 (3)	0.4013 (3)	0.33697 (15)	0.0281 (9)	
H25A	0.1947	0.4044	0.3374	0.034*	
H25B	0.0879	0.3893	0.3763	0.034*	
C26	0.1896 (3)	0.6430 (3)	0.28286 (17)	0.0324 (9)	
H26A	0.2343	0.6648	0.2505	0.049*	
H26B	0.2346	0.6414	0.3174	0.049*	
H26C	0.1289	0.6898	0.2882	0.049*	
C27	0.0374 (3)	0.5723 (3)	0.36052 (16)	0.0305 (9)	
H27A	0.0071	0.6336	0.3440	0.046*	
H27B	0.1035	0.5885	0.3822	0.046*	
H27C	−0.0165	0.5418	0.3860	0.046*	
C28	−0.1281 (3)	0.4260 (3)	0.31389 (18)	0.0323 (9)	
H28A	−0.1639	0.4821	0.3331	0.048*	
H28B	−0.1003	0.3790	0.3424	0.048*	
H28C	−0.1809	0.3918	0.2893	0.048*	
C29	−0.0772 (3)	0.5471 (3)	0.23804 (16)	0.0298 (8)	
H29A	−0.0180	0.5734	0.2143	0.045*	
H29B	−0.1080	0.6013	0.2611	0.045*	
H29C	−0.1343	0.5189	0.2136	0.045*	
C30	0.1413 (3)	0.3465 (2)	0.16380 (14)	0.0206 (7)	
C31	0.0680 (3)	0.3424 (2)	0.11749 (15)	0.0233 (8)	
C32	0.0868 (3)	0.2706 (3)	0.07487 (16)	0.0277 (9)	
H32A	0.0400	0.2690	0.0426	0.033*	
C33	0.1708 (3)	0.2025 (3)	0.07838 (15)	0.0254 (8)	
C34	0.2393 (3)	0.2066 (2)	0.12488 (15)	0.0237 (8)	
H34A	0.2961	0.1587	0.1282	0.028*	
C35	0.2278 (3)	0.2796 (2)	0.16770 (15)	0.0205 (7)	
C36	−0.0318 (3)	0.4082 (3)	0.11195 (18)	0.0336 (9)	
H36A	−0.0504	0.4160	0.0716	0.050*	
H36B	−0.0164	0.4739	0.1285	0.050*	
H36C	−0.0931	0.3773	0.1321	0.050*	
C37	0.1862 (4)	0.1246 (3)	0.03244 (18)	0.0391 (10)	
H37A	0.2363	0.1503	0.0034	0.059*	
H37B	0.1155	0.1094	0.0149	0.059*	
H37C	0.2167	0.0637	0.0493	0.059*	
C38	0.3117 (3)	0.2797 (3)	0.21466 (16)	0.0264 (8)	
H38A	0.2906	0.3282	0.2440	0.040*	
H38B	0.3831	0.2981	0.1989	0.040*	
H38C	0.3161	0.2129	0.2316	0.040*	

### (I) Diiodidobis[(1S,5S)-4-mesityl-1,2,8,8-tetramethyl-2,4-diazabicyclo[3.2.1]octan-3-ylidene-κC3]palladium(IV) Atomic displacement parameters (Å^2^)

**Table:**

	*U*^11^	*U*^22^	*U*^33^	*U*^12^	*U*^13^	*U*^23^
Pd1	0.01775 (12)	0.01382 (10)	0.01575 (12)	−0.00085 (10)	0.00230 (10)	0.00026 (10)
I1	0.02852 (13)	0.03119 (12)	0.02623 (13)	0.00866 (10)	0.00307 (11)	0.00985 (10)
I2	0.02637 (12)	0.03942 (13)	0.02285 (12)	−0.00250 (10)	−0.00341 (10)	0.00155 (10)
N1	0.0327 (17)	0.0148 (13)	0.0271 (17)	−0.0048 (13)	0.0138 (15)	−0.0005 (12)
N2	0.0164 (13)	0.0155 (12)	0.0159 (14)	−0.0031 (11)	0.0017 (11)	0.0017 (11)
N3	0.0188 (14)	0.0161 (13)	0.0210 (15)	−0.0025 (12)	0.0039 (12)	−0.0026 (11)
N4	0.0202 (15)	0.0166 (13)	0.0154 (14)	−0.0014 (11)	0.0031 (12)	−0.0006 (11)
C1	0.0198 (18)	0.0160 (15)	0.0185 (17)	−0.0026 (13)	−0.0001 (14)	−0.0018 (13)
C2	0.030 (2)	0.0177 (16)	0.027 (2)	−0.0072 (15)	0.0128 (16)	0.0010 (15)
C3	0.022 (2)	0.0287 (18)	0.0203 (18)	−0.0123 (15)	0.0009 (15)	0.0006 (15)
C4	0.0160 (17)	0.0190 (15)	0.0239 (19)	−0.0001 (13)	0.0061 (14)	0.0015 (14)
C5	0.025 (2)	0.0240 (18)	0.027 (2)	0.0012 (15)	−0.0006 (16)	0.0084 (15)
C6	0.026 (2)	0.0225 (16)	0.0169 (17)	−0.0031 (15)	0.0004 (15)	0.0008 (14)
C7	0.048 (3)	0.0182 (17)	0.048 (3)	−0.0082 (17)	0.025 (2)	−0.0108 (17)
C8	0.056 (3)	0.0200 (18)	0.049 (3)	−0.0101 (19)	0.022 (2)	−0.0015 (18)
C9	0.027 (2)	0.041 (2)	0.028 (2)	−0.0195 (17)	0.0021 (17)	0.0031 (18)
C10	0.039 (2)	0.045 (2)	0.022 (2)	−0.023 (2)	−0.0064 (19)	0.0019 (17)
C11	0.0235 (18)	0.0167 (15)	0.0199 (18)	0.0043 (13)	0.0082 (14)	0.0002 (13)
C12	0.0208 (19)	0.0214 (17)	0.0210 (18)	−0.0003 (14)	0.0096 (15)	−0.0014 (14)
C13	0.036 (2)	0.0202 (17)	0.027 (2)	−0.0044 (16)	0.0127 (18)	−0.0043 (15)
C14	0.047 (3)	0.0185 (18)	0.035 (2)	0.0064 (18)	0.017 (2)	0.0040 (16)
C15	0.039 (2)	0.0271 (19)	0.038 (2)	0.0154 (18)	0.008 (2)	0.0077 (17)
C16	0.027 (2)	0.0271 (18)	0.0230 (19)	0.0059 (16)	0.0067 (15)	0.0029 (15)
C17	0.026 (2)	0.0270 (17)	0.0238 (19)	−0.0057 (16)	−0.0012 (15)	−0.0055 (15)
C18	0.077 (4)	0.0167 (18)	0.052 (3)	0.014 (2)	0.013 (3)	0.0001 (19)
C19	0.028 (2)	0.039 (2)	0.032 (2)	0.0083 (17)	−0.0049 (18)	0.0059 (17)
C20	0.0168 (17)	0.0154 (14)	0.0170 (16)	0.0035 (13)	−0.0013 (13)	0.0043 (12)
C21	0.0234 (18)	0.0223 (16)	0.0200 (18)	0.0034 (15)	0.0071 (15)	0.0047 (14)
C22	0.0189 (17)	0.0215 (17)	0.0246 (18)	0.0021 (14)	0.0073 (14)	0.0036 (14)
C23	0.0230 (18)	0.0177 (15)	0.0245 (18)	−0.0029 (14)	0.0080 (15)	−0.0016 (15)
C24	0.035 (2)	0.0192 (16)	0.030 (2)	0.0017 (16)	0.0156 (18)	0.0035 (15)
C25	0.037 (2)	0.0299 (18)	0.0179 (19)	0.0077 (16)	0.0066 (16)	0.0051 (15)
C26	0.036 (2)	0.0238 (18)	0.037 (2)	−0.0051 (17)	0.0133 (19)	−0.0104 (16)
C27	0.037 (2)	0.0278 (19)	0.026 (2)	0.0029 (17)	0.0149 (18)	−0.0014 (16)
C28	0.026 (2)	0.032 (2)	0.039 (2)	−0.0007 (16)	0.0145 (18)	0.0040 (17)
C29	0.0241 (19)	0.0317 (18)	0.034 (2)	0.0069 (16)	0.0005 (17)	0.0053 (18)
C30	0.0258 (19)	0.0158 (15)	0.0202 (18)	−0.0061 (14)	0.0043 (15)	−0.0022 (13)
C31	0.0247 (19)	0.0241 (17)	0.0212 (18)	−0.0071 (15)	0.0027 (16)	0.0010 (14)
C32	0.033 (2)	0.0299 (19)	0.0206 (19)	−0.0148 (17)	−0.0014 (16)	−0.0024 (15)
C33	0.031 (2)	0.0255 (17)	0.0194 (18)	−0.0140 (16)	0.0087 (16)	−0.0043 (14)
C34	0.027 (2)	0.0179 (16)	0.026 (2)	−0.0053 (14)	0.0080 (16)	−0.0013 (14)
C35	0.0239 (19)	0.0185 (15)	0.0192 (18)	−0.0076 (13)	0.0061 (15)	0.0003 (14)
C36	0.028 (2)	0.040 (2)	0.032 (2)	−0.0013 (17)	−0.0080 (18)	−0.0014 (18)
C37	0.046 (3)	0.037 (2)	0.035 (2)	−0.014 (2)	0.004 (2)	−0.0147 (19)
C38	0.032 (2)	0.0250 (17)	0.0223 (19)	0.0077 (16)	0.0029 (16)	−0.0007 (15)

### (I) Diiodidobis[(1S,5S)-4-mesityl-1,2,8,8-tetramethyl-2,4-diazabicyclo[3.2.1]octan-3-ylidene-κC3]palladium(IV) Geometric parameters (Å, º)

**Table:**

Pd1—C1	2.070 (3)	C17—H17B	0.9800
Pd1—C20	2.079 (3)	C17—H17C	0.9800
Pd1—I1	2.6334 (4)	C18—H18A	0.9800
Pd1—I2	2.6360 (4)	C18—H18B	0.9800
N1—C1	1.351 (4)	C18—H18C	0.9800
N1—C7	1.454 (5)	C19—H19A	0.9800
N1—C2	1.496 (4)	C19—H19B	0.9800
N2—C1	1.354 (4)	C19—H19C	0.9800
N2—C11	1.448 (4)	C21—C27	1.514 (5)
N2—C4	1.491 (4)	C21—C22	1.537 (5)
N3—C20	1.343 (4)	C21—C25	1.548 (5)
N3—C26	1.468 (4)	C22—C29	1.534 (5)
N3—C21	1.505 (4)	C22—C23	1.538 (5)
N4—C20	1.333 (4)	C22—C28	1.544 (5)
N4—C30	1.451 (4)	C23—C24	1.540 (5)
N4—C23	1.503 (4)	C23—H23A	1.0000
C2—C5	1.541 (5)	C24—C25	1.545 (5)
C2—C8	1.541 (5)	C24—H24A	0.9900
C2—C3	1.545 (5)	C24—H24B	0.9900
C3—C10	1.522 (5)	C25—H25A	0.9900
C3—C4	1.528 (5)	C25—H25B	0.9900
C3—C9	1.535 (5)	C26—H26A	0.9800
C4—C6	1.529 (5)	C26—H26B	0.9800
C4—H4A	1.0000	C26—H26C	0.9800
C5—C6	1.547 (5)	C27—H27A	0.9800
C5—H5A	0.9900	C27—H27B	0.9800
C5—H5B	0.9900	C27—H27C	0.9800
C6—H6A	0.9900	C28—H28A	0.9800
C6—H6B	0.9900	C28—H28B	0.9800
C7—H7A	0.9800	C28—H28C	0.9800
C7—H7B	0.9800	C29—H29A	0.9800
C7—H7C	0.9800	C29—H29B	0.9800
C8—H8A	0.9800	C29—H29C	0.9800
C8—H8B	0.9800	C30—C35	1.390 (5)
C8—H8C	0.9800	C30—C31	1.410 (5)
C9—H9A	0.9800	C31—C32	1.406 (5)
C9—H9B	0.9800	C31—C36	1.512 (5)
C9—H9C	0.9800	C32—C33	1.377 (5)
C10—H10A	0.9800	C32—H32A	0.9500
C10—H10B	0.9800	C33—C34	1.377 (5)
C10—H10C	0.9800	C33—C37	1.511 (5)
C11—C12	1.398 (5)	C34—C35	1.408 (5)
C11—C16	1.409 (5)	C34—H34A	0.9500
C12—C13	1.401 (5)	C35—C38	1.507 (5)
C12—C17	1.503 (5)	C36—H36A	0.9800
C13—C14	1.384 (6)	C36—H36B	0.9800
C13—H13A	0.9500	C36—H36C	0.9800
C14—C15	1.383 (6)	C37—H37A	0.9800
C14—C18	1.517 (5)	C37—H37B	0.9800
C15—C16	1.388 (5)	C37—H37C	0.9800
C15—H15A	0.9500	C38—H38A	0.9800
C16—C19	1.503 (5)	C38—H38B	0.9800
C17—H17A	0.9800	C38—H38C	0.9800
			
C1—Pd1—C20	178.32 (12)	C14—C18—H18C	109.5
C1—Pd1—I1	91.63 (9)	H18A—C18—H18C	109.5
C20—Pd1—I1	87.63 (8)	H18B—C18—H18C	109.5
C1—Pd1—I2	89.05 (9)	C16—C19—H19A	109.5
C20—Pd1—I2	91.23 (9)	C16—C19—H19B	109.5
I1—Pd1—I2	163.275 (13)	H19A—C19—H19B	109.5
C1—N1—C7	119.8 (3)	C16—C19—H19C	109.5
C1—N1—C2	121.2 (3)	H19A—C19—H19C	109.5
C7—N1—C2	118.9 (3)	H19B—C19—H19C	109.5
C1—N2—C11	123.3 (3)	N4—C20—N3	116.3 (3)
C1—N2—C4	123.1 (3)	N4—C20—Pd1	123.9 (2)
C11—N2—C4	113.3 (3)	N3—C20—Pd1	119.8 (2)
C20—N3—C26	119.7 (3)	N3—C21—C27	112.4 (3)
C20—N3—C21	122.2 (3)	N3—C21—C22	105.8 (3)
C26—N3—C21	117.9 (3)	C27—C21—C22	114.6 (3)
C20—N4—C30	124.1 (3)	N3—C21—C25	107.9 (3)
C20—N4—C23	122.4 (3)	C27—C21—C25	111.7 (3)
C30—N4—C23	113.1 (3)	C22—C21—C25	103.9 (3)
N1—C1—N2	115.7 (3)	C29—C22—C21	113.3 (3)
N1—C1—Pd1	120.7 (2)	C29—C22—C23	115.6 (3)
N2—C1—Pd1	123.5 (2)	C21—C22—C23	97.5 (3)
N1—C2—C5	108.8 (3)	C29—C22—C28	107.8 (3)
N1—C2—C8	112.2 (3)	C21—C22—C28	113.1 (3)
C5—C2—C8	111.2 (3)	C23—C22—C28	109.5 (3)
N1—C2—C3	105.7 (3)	N4—C23—C22	110.4 (3)
C5—C2—C3	104.1 (3)	N4—C23—C24	107.9 (3)
C8—C2—C3	114.3 (3)	C22—C23—C24	103.4 (3)
C10—C3—C4	115.4 (3)	N4—C23—H23A	111.6
C10—C3—C9	107.8 (3)	C22—C23—H23A	111.6
C4—C3—C9	109.9 (3)	C24—C23—H23A	111.6
C10—C3—C2	113.2 (3)	C23—C24—C25	102.0 (3)
C4—C3—C2	97.4 (3)	C23—C24—H24A	111.4
C9—C3—C2	113.1 (3)	C25—C24—H24A	111.4
N2—C4—C3	110.0 (3)	C23—C24—H24B	111.4
N2—C4—C6	108.2 (3)	C25—C24—H24B	111.4
C3—C4—C6	103.3 (3)	H24A—C24—H24B	109.2
N2—C4—H4A	111.6	C24—C25—C21	106.2 (3)
C3—C4—H4A	111.6	C24—C25—H25A	110.5
C6—C4—H4A	111.6	C21—C25—H25A	110.5
C2—C5—C6	106.0 (3)	C24—C25—H25B	110.5
C2—C5—H5A	110.5	C21—C25—H25B	110.5
C6—C5—H5A	110.5	H25A—C25—H25B	108.7
C2—C5—H5B	110.5	N3—C26—H26A	109.5
C6—C5—H5B	110.5	N3—C26—H26B	109.5
H5A—C5—H5B	108.7	H26A—C26—H26B	109.5
C4—C6—C5	102.0 (3)	N3—C26—H26C	109.5
C4—C6—H6A	111.4	H26A—C26—H26C	109.5
C5—C6—H6A	111.4	H26B—C26—H26C	109.5
C4—C6—H6B	111.4	C21—C27—H27A	109.5
C5—C6—H6B	111.4	C21—C27—H27B	109.5
H6A—C6—H6B	109.2	H27A—C27—H27B	109.5
N1—C7—H7A	109.5	C21—C27—H27C	109.5
N1—C7—H7B	109.5	H27A—C27—H27C	109.5
H7A—C7—H7B	109.5	H27B—C27—H27C	109.5
N1—C7—H7C	109.5	C22—C28—H28A	109.5
H7A—C7—H7C	109.5	C22—C28—H28B	109.5
H7B—C7—H7C	109.5	H28A—C28—H28B	109.5
C2—C8—H8A	109.5	C22—C28—H28C	109.5
C2—C8—H8B	109.5	H28A—C28—H28C	109.5
H8A—C8—H8B	109.5	H28B—C28—H28C	109.5
C2—C8—H8C	109.5	C22—C29—H29A	109.5
H8A—C8—H8C	109.5	C22—C29—H29B	109.5
H8B—C8—H8C	109.5	H29A—C29—H29B	109.5
C3—C9—H9A	109.5	C22—C29—H29C	109.5
C3—C9—H9B	109.5	H29A—C29—H29C	109.5
H9A—C9—H9B	109.5	H29B—C29—H29C	109.5
C3—C9—H9C	109.5	C35—C30—C31	120.7 (3)
H9A—C9—H9C	109.5	C35—C30—N4	121.1 (3)
H9B—C9—H9C	109.5	C31—C30—N4	117.7 (3)
C3—C10—H10A	109.5	C32—C31—C30	118.1 (3)
C3—C10—H10B	109.5	C32—C31—C36	118.0 (3)
H10A—C10—H10B	109.5	C30—C31—C36	123.9 (3)
C3—C10—H10C	109.5	C33—C32—C31	122.1 (4)
H10A—C10—H10C	109.5	C33—C32—H32A	118.9
H10B—C10—H10C	109.5	C31—C32—H32A	118.9
C12—C11—C16	120.7 (3)	C32—C33—C34	118.4 (3)
C12—C11—N2	120.9 (3)	C32—C33—C37	120.5 (4)
C16—C11—N2	117.7 (3)	C34—C33—C37	121.1 (4)
C11—C12—C13	118.1 (3)	C33—C34—C35	122.2 (3)
C11—C12—C17	124.1 (3)	C33—C34—H34A	118.9
C13—C12—C17	117.8 (3)	C35—C34—H34A	118.9
C14—C13—C12	122.5 (4)	C30—C35—C34	118.4 (3)
C14—C13—H13A	118.7	C30—C35—C38	124.6 (3)
C12—C13—H13A	118.7	C34—C35—C38	117.0 (3)
C15—C14—C13	117.7 (3)	C31—C36—H36A	109.5
C15—C14—C18	120.4 (4)	C31—C36—H36B	109.5
C13—C14—C18	121.9 (4)	H36A—C36—H36B	109.5
C14—C15—C16	122.7 (4)	C31—C36—H36C	109.5
C14—C15—H15A	118.7	H36A—C36—H36C	109.5
C16—C15—H15A	118.7	H36B—C36—H36C	109.5
C15—C16—C11	118.3 (4)	C33—C37—H37A	109.5
C15—C16—C19	118.4 (3)	C33—C37—H37B	109.5
C11—C16—C19	123.3 (3)	H37A—C37—H37B	109.5
C12—C17—H17A	109.5	C33—C37—H37C	109.5
C12—C17—H17B	109.5	H37A—C37—H37C	109.5
H17A—C17—H17B	109.5	H37B—C37—H37C	109.5
C12—C17—H17C	109.5	C35—C38—H38A	109.5
H17A—C17—H17C	109.5	C35—C38—H38B	109.5
H17B—C17—H17C	109.5	H38A—C38—H38B	109.5
C14—C18—H18A	109.5	C35—C38—H38C	109.5
C14—C18—H18B	109.5	H38A—C38—H38C	109.5
H18A—C18—H18B	109.5	H38B—C38—H38C	109.5
			
C7—N1—C1—N2	−166.4 (3)	C30—N4—C20—N3	−172.2 (3)
C2—N1—C1—N2	9.1 (5)	C23—N4—C20—N3	0.3 (4)
C7—N1—C1—Pd1	15.6 (5)	C30—N4—C20—Pd1	6.8 (4)
C2—N1—C1—Pd1	−168.9 (3)	C23—N4—C20—Pd1	179.3 (2)
C11—N2—C1—N1	−173.5 (3)	C26—N3—C20—N4	−167.2 (3)
C4—N2—C1—N1	1.0 (5)	C21—N3—C20—N4	8.1 (4)
C11—N2—C1—Pd1	4.5 (5)	C26—N3—C20—Pd1	13.7 (4)
C4—N2—C1—Pd1	179.0 (2)	C21—N3—C20—Pd1	−170.9 (2)
I1—Pd1—C1—N1	61.3 (3)	I1—Pd1—C20—N4	79.8 (3)
I2—Pd1—C1—N1	−102.0 (3)	I2—Pd1—C20—N4	−116.9 (3)
I1—Pd1—C1—N2	−116.5 (3)	I1—Pd1—C20—N3	−101.2 (2)
I2—Pd1—C1—N2	80.2 (3)	I2—Pd1—C20—N3	62.1 (2)
C1—N1—C2—C5	62.4 (4)	C20—N3—C21—C27	−172.7 (3)
C7—N1—C2—C5	−122.1 (4)	C26—N3—C21—C27	2.7 (4)
C1—N1—C2—C8	−174.2 (4)	C20—N3—C21—C22	−47.0 (4)
C7—N1—C2—C8	1.4 (5)	C26—N3—C21—C22	128.4 (3)
C1—N1—C2—C3	−48.9 (4)	C20—N3—C21—C25	63.7 (4)
C7—N1—C2—C3	126.6 (4)	C26—N3—C21—C25	−120.8 (3)
N1—C2—C3—C10	−50.4 (4)	N3—C21—C22—C29	−52.7 (3)
C5—C2—C3—C10	−164.9 (3)	C27—C21—C22—C29	71.7 (4)
C8—C2—C3—C10	73.6 (4)	C25—C21—C22—C29	−166.2 (3)
N1—C2—C3—C4	71.3 (3)	N3—C21—C22—C23	69.4 (3)
C5—C2—C3—C4	−43.2 (3)	C27—C21—C22—C23	−166.2 (3)
C8—C2—C3—C4	−164.7 (3)	C25—C21—C22—C23	−44.1 (3)
N1—C2—C3—C9	−173.3 (3)	N3—C21—C22—C28	−175.7 (3)
C5—C2—C3—C9	72.1 (4)	C27—C21—C22—C28	−51.3 (4)
C8—C2—C3—C9	−49.3 (4)	C25—C21—C22—C28	70.8 (3)
C1—N2—C4—C3	30.3 (4)	C20—N4—C23—C22	31.4 (4)
C11—N2—C4—C3	−154.7 (3)	C30—N4—C23—C22	−155.3 (3)
C1—N2—C4—C6	−82.0 (4)	C20—N4—C23—C24	−80.9 (4)
C11—N2—C4—C6	93.0 (3)	C30—N4—C23—C24	92.3 (3)
C10—C3—C4—N2	56.7 (4)	C29—C22—C23—N4	56.6 (4)
C9—C3—C4—N2	178.7 (3)	C21—C22—C23—N4	−63.8 (3)
C2—C3—C4—N2	−63.4 (3)	C28—C22—C23—N4	178.5 (3)
C10—C3—C4—C6	172.0 (3)	C29—C22—C23—C24	171.8 (3)
C9—C3—C4—C6	−65.9 (4)	C21—C22—C23—C24	51.4 (3)
C2—C3—C4—C6	52.0 (3)	C28—C22—C23—C24	−66.3 (4)
N1—C2—C5—C6	−92.5 (3)	N4—C23—C24—C25	78.6 (3)
C8—C2—C5—C6	143.4 (3)	C22—C23—C24—C25	−38.4 (3)
C3—C2—C5—C6	19.9 (3)	C23—C24—C25—C21	9.8 (4)
N2—C4—C6—C5	76.3 (3)	N3—C21—C25—C24	−90.1 (3)
C3—C4—C6—C5	−40.3 (3)	C27—C21—C25—C24	145.9 (3)
C2—C5—C6—C4	12.0 (3)	C22—C21—C25—C24	21.9 (4)
C1—N2—C11—C12	76.8 (4)	C20—N4—C30—C35	74.6 (4)
C4—N2—C11—C12	−98.3 (4)	C23—N4—C30—C35	−98.5 (4)
C1—N2—C11—C16	−112.5 (4)	C20—N4—C30—C31	−112.9 (4)
C4—N2—C11—C16	72.5 (4)	C23—N4—C30—C31	74.0 (4)
C16—C11—C12—C13	−0.5 (5)	C35—C30—C31—C32	−1.6 (5)
N2—C11—C12—C13	170.0 (3)	N4—C30—C31—C32	−174.1 (3)
C16—C11—C12—C17	179.8 (3)	C35—C30—C31—C36	176.1 (3)
N2—C11—C12—C17	−9.7 (5)	N4—C30—C31—C36	3.6 (5)
C11—C12—C13—C14	0.8 (5)	C30—C31—C32—C33	2.8 (5)
C17—C12—C13—C14	−179.5 (3)	C36—C31—C32—C33	−175.1 (3)
C12—C13—C14—C15	0.3 (6)	C31—C32—C33—C34	−1.1 (5)
C12—C13—C14—C18	179.7 (4)	C31—C32—C33—C37	178.2 (3)
C13—C14—C15—C16	−1.7 (6)	C32—C33—C34—C35	−1.8 (5)
C18—C14—C15—C16	178.9 (4)	C37—C33—C34—C35	178.8 (3)
C14—C15—C16—C11	2.0 (6)	C31—C30—C35—C34	−1.1 (5)
C14—C15—C16—C19	−176.8 (4)	N4—C30—C35—C34	171.1 (3)
C12—C11—C16—C15	−0.8 (5)	C31—C30—C35—C38	178.4 (3)
N2—C11—C16—C15	−171.6 (3)	N4—C30—C35—C38	−9.3 (5)
C12—C11—C16—C19	177.9 (3)	C33—C34—C35—C30	2.9 (5)
N2—C11—C16—C19	7.1 (5)	C33—C34—C35—C38	−176.6 (3)

### (I) Diiodidobis[(1S,5S)-4-mesityl-1,2,8,8-tetramethyl-2,4-diazabicyclo[3.2.1]octan-3-ylidene-κC3]palladium(IV) Hydrogen-bond geometry (Å, º)

**Table:**

*D*—H···*A*	*D*—H	H···*A*	*D*···*A*	*D*—H···*A*
C5—H5*A*···I1^i^	0.99	3.22	4.131 (5)	154

Symmetry code: (i) *x*+1/2, −*y*+3/2, −*z*.

### (II) Dichlorido[(1S,5S)-4-mesityl-1,2,8,8-tetramethyl-2,4-diazabicyclo[3.2.1]octan-3-ylidene-κC3](triphenylphosphane-κP)palladium(IV) Crystal data

**Table:**

[Pd(C_19_H_28_N_2_)Cl_2_(C_18_H_15_P)]	*F*(000) = 748
*M**_r_* = 724.00	*D*_x_ = 1.393 Mg m^−^^3^
Monoclinic, *P*2_1_	Mo *K*α radiation, λ = 0.71073 Å
*a* = 10.987 (3) Å	Cell parameters from 3348 reflections
*b* = 9.568 (2) Å	θ = 2.5–27.0°
*c* = 17.211 (4) Å	µ = 0.77 mm^−^^1^
β = 107.478 (4)°	*T* = 130 K
*V* = 1725.7 (7) Å^3^	Plate, colourless
*Z* = 2	0.48 × 0.20 × 0.01 mm

### (II) Dichlorido[(1S,5S)-4-mesityl-1,2,8,8-tetramethyl-2,4-diazabicyclo[3.2.1]octan-3-ylidene-κC3](triphenylphosphane-κP)palladium(IV) Data collection

**Table:**

Bruker SMART APEX diffractometer	8099 independent reflections
Radiation source: sealed tube	6415 reflections with *I* > 2σ(*I*)
Graphite monochromator	*R*_int_ = 0.089
φ and ω scans	θ_max_ = 27.9°, θ_min_ = 1.2°
Absorption correction: multi-scan (*SADABS*; Bruker, 2002)	*h* = −13→14
*T*_min_ = 0.710, *T*_max_ = 0.992	*k* = −12→12
13973 measured reflections	*l* = −22→22

### (II) Dichlorido[(1S,5S)-4-mesityl-1,2,8,8-tetramethyl-2,4-diazabicyclo[3.2.1]octan-3-ylidene-κC3](triphenylphosphane-κP)palladium(IV) Refinement

**Table:**

Refinement on *F*^2^	Secondary atom site location: difference Fourier map
Least-squares matrix: full	Hydrogen site location: difference Fourier map
*R*[*F*^2^ > 2σ(*F*^2^)] = 0.053	H-atom parameters constrained
*wR*(*F*^2^) = 0.113	*w* = 1/[σ^2^(*F*_o_^2^) + (0.0399*P*)^2^] where *P* = (*F*_o_^2^ + 2*F*_c_^2^)/3
*S* = 0.92	(Δ/σ)_max_ = 0.001
8099 reflections	Δρ_max_ = 1.00 e Å^−^^3^
393 parameters	Δρ_min_ = −1.60 e Å^−^^3^
1 restraint	Absolute structure: Flack *x* determined using 2367 quotients [(*I*^+^)-(*I*^-^)]/[(*I*^+^)+(*I*^-^)] (Parsons & Flack, 2004)
Primary atom site location: structure-invariant direct methods	Absolute structure parameter: −0.02 (4)

### (II) Dichlorido[(1S,5S)-4-mesityl-1,2,8,8-tetramethyl-2,4-diazabicyclo[3.2.1]octan-3-ylidene-κC3](triphenylphosphane-κP)palladium(IV) Special details

**Table:**

Geometry. All e.s.d.'s (except the e.s.d. in the dihedral angle between two l.s. planes) are estimated using the full covariance matrix. The cell e.s.d.'s are taken into account individually in the estimation of e.s.d.'s in distances, angles and torsion angles; correlations between e.s.d.'s in cell parameters are only used when they are defined by crystal symmetry. An approximate (isotropic) treatment of cell e.s.d.'s is used for estimating e.s.d.'s involving l.s. planes.

### (II) Dichlorido[(1S,5S)-4-mesityl-1,2,8,8-tetramethyl-2,4-diazabicyclo[3.2.1]octan-3-ylidene-κC3](triphenylphosphane-κP)palladium(IV) Fractional atomic coordinates and isotropic or equivalent isotropic displacement parameters (Å^2^)

**Table:**

	*x*	*y*	*z*	*U*_iso_*/*U*_eq_	
Pd1	0.24938 (5)	0.19189 (6)	0.31693 (3)	0.01671 (14)	
Cl1	0.4202 (2)	0.3441 (2)	0.34508 (12)	0.0312 (5)	
Cl2	0.09193 (19)	0.0248 (2)	0.30106 (12)	0.0252 (5)	
P1	0.26931 (18)	0.1278 (2)	0.18920 (12)	0.0185 (4)	
N1	0.2705 (5)	0.1616 (7)	0.4905 (3)	0.0209 (17)	
N2	0.1623 (6)	0.3605 (6)	0.4351 (3)	0.0152 (13)	
C1	0.2245 (6)	0.2440 (8)	0.4266 (4)	0.0168 (16)	
C2	0.3400 (9)	0.0354 (10)	0.4841 (5)	0.032 (2)	
H2A	0.2954	−0.0459	0.4971	0.048*	
H2B	0.4262	0.0404	0.5224	0.048*	
H2C	0.3452	0.0265	0.4285	0.048*	
C3	0.2468 (6)	0.1965 (12)	0.5712 (3)	0.0183 (13)	
C4	0.3261 (8)	0.1040 (9)	0.6390 (5)	0.0260 (18)	
H4A	0.3047	0.0058	0.6253	0.039*	
H4B	0.3082	0.1272	0.6900	0.039*	
H4C	0.4169	0.1192	0.6457	0.039*	
C5	0.1005 (6)	0.1808 (11)	0.5580 (4)	0.0222 (16)	
H5A	0.0629	0.1123	0.5142	0.027*	
H5B	0.0843	0.1488	0.6087	0.027*	
C6	0.0429 (7)	0.3274 (9)	0.5337 (5)	0.0215 (17)	
H6A	−0.0321	0.3231	0.4844	0.026*	
H6B	0.0175	0.3712	0.5786	0.026*	
C7	0.1532 (7)	0.4064 (8)	0.5170 (4)	0.0161 (15)	
H7A	0.1426	0.5099	0.5196	0.019*	
C8	0.2712 (7)	0.3541 (8)	0.5838 (4)	0.0193 (16)	
C9	0.2635 (8)	0.4027 (9)	0.6674 (4)	0.0274 (19)	
H9A	0.2789	0.5037	0.6730	0.041*	
H9B	0.3281	0.3536	0.7106	0.041*	
H9C	0.1785	0.3819	0.6719	0.041*	
C10	0.3999 (7)	0.4017 (9)	0.5759 (5)	0.0240 (18)	
H10A	0.4089	0.5028	0.5847	0.036*	
H10B	0.4043	0.3791	0.5213	0.036*	
H10C	0.4689	0.3535	0.6167	0.036*	
C11	0.0918 (6)	0.4438 (8)	0.3657 (4)	0.0154 (15)	
C12	−0.0267 (7)	0.3981 (8)	0.3150 (4)	0.0183 (15)	
C13	−0.0907 (8)	0.4853 (9)	0.2506 (5)	0.0251 (18)	
H13A	−0.1702	0.4545	0.2151	0.030*	
C14	−0.0449 (8)	0.6143 (9)	0.2354 (4)	0.0238 (17)	
C15	0.0712 (7)	0.6563 (8)	0.2895 (4)	0.0234 (19)	
H15A	0.1043	0.7449	0.2812	0.028*	
C16	0.1408 (7)	0.5763 (8)	0.3545 (5)	0.0207 (16)	
C17	−0.0946 (7)	0.2674 (8)	0.3277 (5)	0.0264 (18)	
H17A	−0.1720	0.2927	0.3415	0.040*	
H17B	−0.0382	0.2126	0.3723	0.040*	
H17C	−0.1176	0.2116	0.2776	0.040*	
C18	−0.1167 (8)	0.7054 (13)	0.1664 (5)	0.038 (2)	
H18A	−0.0961	0.6784	0.1169	0.057*	
H18B	−0.0925	0.8032	0.1795	0.057*	
H18C	−0.2085	0.6944	0.1576	0.057*	
C19	0.2627 (8)	0.6319 (9)	0.4105 (5)	0.0268 (18)	
H19A	0.2817	0.7227	0.3904	0.040*	
H19B	0.3322	0.5664	0.4125	0.040*	
H19C	0.2542	0.6431	0.4652	0.040*	
C21	0.1114 (7)	0.1308 (9)	0.1132 (4)	0.0212 (16)	
C22	0.0823 (9)	0.0484 (10)	0.0436 (5)	0.032 (2)	
H22A	0.1436	−0.0154	0.0354	0.038*	
C23	−0.0370 (9)	0.0598 (11)	−0.0140 (5)	0.040 (2)	
H23A	−0.0570	0.0048	−0.0621	0.048*	
C24	−0.1263 (8)	0.1510 (10)	−0.0012 (6)	0.040 (3)	
H24A	−0.2083	0.1575	−0.0401	0.048*	
C25	−0.0972 (8)	0.2320 (10)	0.0672 (5)	0.037 (2)	
H25A	−0.1586	0.2962	0.0748	0.045*	
C26	0.0210 (7)	0.2219 (9)	0.1257 (5)	0.030 (2)	
H26A	0.0397	0.2768	0.1738	0.035*	
C31	0.3659 (7)	0.2364 (8)	0.1431 (4)	0.0216 (17)	
C32	0.3177 (8)	0.3112 (9)	0.0720 (5)	0.0268 (18)	
H32A	0.2282	0.3117	0.0459	0.032*	
C33	0.3971 (9)	0.3857 (10)	0.0379 (5)	0.033 (2)	
H33A	0.3626	0.4372	−0.0110	0.039*	
C34	0.5277 (9)	0.3841 (11)	0.0760 (6)	0.039 (2)	
H34A	0.5833	0.4341	0.0531	0.047*	
C35	0.5761 (8)	0.3100 (11)	0.1468 (6)	0.038 (2)	
H35A	0.6658	0.3090	0.1721	0.046*	
C36	0.4984 (8)	0.2372 (9)	0.1822 (5)	0.032 (2)	
H36A	0.5336	0.1884	0.2319	0.039*	
C41	0.3339 (7)	−0.0452 (9)	0.1821 (4)	0.0198 (16)	
C42	0.3127 (7)	−0.1522 (9)	0.2321 (5)	0.0258 (18)	
H42A	0.2665	−0.1322	0.2694	0.031*	
C43	0.3577 (7)	−0.2863 (10)	0.2282 (4)	0.026 (2)	
H43A	0.3408	−0.3582	0.2617	0.032*	
C44	0.4271 (6)	−0.3147 (13)	0.1755 (4)	0.0269 (16)	
H44A	0.4595	−0.4062	0.1735	0.032*	
C45	0.4497 (8)	−0.2128 (9)	0.1261 (5)	0.0291 (19)	
H45A	0.4976	−0.2340	0.0899	0.035*	
C46	0.4038 (8)	−0.0798 (10)	0.1283 (5)	0.0280 (19)	
H46A	0.4195	−0.0103	0.0931	0.034*	

### (II) Dichlorido[(1S,5S)-4-mesityl-1,2,8,8-tetramethyl-2,4-diazabicyclo[3.2.1]octan-3-ylidene-κC3](triphenylphosphane-κP)palladium(IV) Atomic displacement parameters (Å^2^)

**Table:**

	*U*^11^	*U*^22^	*U*^33^	*U*^12^	*U*^13^	*U*^23^
Pd1	0.0131 (2)	0.0177 (3)	0.0243 (2)	−0.0021 (3)	0.01319 (18)	−0.0018 (3)
Cl1	0.0278 (11)	0.0357 (13)	0.0383 (11)	−0.0194 (10)	0.0222 (9)	−0.0155 (10)
Cl2	0.0216 (10)	0.0206 (11)	0.0409 (11)	−0.0070 (8)	0.0206 (9)	−0.0044 (9)
P1	0.0142 (9)	0.0201 (10)	0.0252 (9)	−0.0009 (8)	0.0120 (8)	−0.0008 (9)
N1	0.015 (3)	0.026 (5)	0.026 (3)	0.004 (3)	0.013 (2)	0.003 (3)
N2	0.014 (3)	0.014 (3)	0.021 (3)	0.000 (3)	0.011 (2)	−0.001 (2)
C1	0.008 (3)	0.014 (4)	0.031 (4)	−0.006 (3)	0.008 (3)	−0.011 (3)
C2	0.038 (5)	0.031 (5)	0.033 (4)	0.012 (4)	0.020 (4)	0.000 (4)
C3	0.016 (3)	0.023 (4)	0.021 (3)	−0.007 (5)	0.012 (2)	0.001 (5)
C4	0.022 (4)	0.026 (5)	0.033 (4)	0.006 (4)	0.013 (3)	0.004 (4)
C5	0.013 (3)	0.025 (4)	0.033 (3)	−0.001 (4)	0.015 (3)	0.004 (5)
C6	0.018 (4)	0.025 (5)	0.027 (4)	−0.001 (3)	0.015 (3)	−0.001 (3)
C7	0.013 (3)	0.017 (4)	0.023 (3)	0.002 (3)	0.013 (3)	−0.006 (3)
C8	0.016 (4)	0.023 (4)	0.023 (3)	−0.002 (3)	0.011 (3)	−0.004 (3)
C9	0.026 (4)	0.030 (5)	0.029 (4)	0.003 (4)	0.012 (4)	−0.002 (4)
C10	0.013 (4)	0.028 (5)	0.033 (4)	−0.006 (3)	0.009 (3)	−0.002 (4)
C11	0.009 (3)	0.019 (4)	0.022 (3)	−0.002 (3)	0.010 (3)	−0.005 (3)
C12	0.012 (3)	0.019 (4)	0.028 (4)	0.000 (3)	0.013 (3)	−0.004 (3)
C13	0.016 (4)	0.029 (5)	0.030 (4)	0.005 (3)	0.008 (3)	−0.003 (4)
C14	0.029 (4)	0.025 (5)	0.023 (4)	0.003 (4)	0.016 (3)	0.001 (3)
C15	0.026 (4)	0.017 (5)	0.035 (4)	0.001 (3)	0.020 (3)	0.003 (3)
C16	0.018 (4)	0.015 (4)	0.034 (4)	0.002 (3)	0.016 (3)	0.000 (3)
C17	0.016 (4)	0.021 (5)	0.043 (5)	−0.004 (3)	0.009 (3)	−0.004 (4)
C18	0.038 (4)	0.037 (6)	0.040 (4)	0.012 (6)	0.015 (4)	0.008 (5)
C19	0.028 (4)	0.023 (4)	0.033 (4)	−0.009 (4)	0.015 (3)	−0.002 (3)
C21	0.017 (4)	0.026 (4)	0.024 (4)	−0.006 (3)	0.011 (3)	0.003 (3)
C22	0.028 (5)	0.031 (5)	0.033 (4)	−0.002 (4)	0.006 (4)	−0.008 (4)
C23	0.031 (5)	0.045 (6)	0.037 (5)	−0.003 (5)	−0.002 (4)	−0.007 (4)
C24	0.021 (4)	0.047 (7)	0.047 (5)	−0.003 (4)	0.000 (4)	0.005 (4)
C25	0.022 (4)	0.042 (7)	0.050 (5)	0.008 (4)	0.015 (4)	0.007 (4)
C26	0.023 (4)	0.037 (7)	0.033 (4)	0.000 (4)	0.015 (3)	0.004 (4)
C31	0.019 (4)	0.021 (4)	0.029 (4)	−0.001 (3)	0.014 (3)	−0.002 (3)
C32	0.024 (4)	0.028 (5)	0.031 (4)	−0.004 (4)	0.013 (3)	−0.001 (4)
C33	0.045 (6)	0.028 (5)	0.034 (4)	−0.007 (4)	0.025 (4)	0.000 (4)
C34	0.039 (6)	0.041 (6)	0.050 (5)	−0.016 (5)	0.032 (5)	−0.007 (5)
C35	0.023 (4)	0.049 (6)	0.048 (5)	−0.014 (4)	0.020 (4)	−0.005 (5)
C36	0.021 (4)	0.046 (6)	0.033 (4)	−0.008 (4)	0.015 (3)	0.001 (4)
C41	0.010 (3)	0.024 (4)	0.026 (4)	−0.001 (3)	0.006 (3)	0.000 (3)
C42	0.020 (4)	0.029 (5)	0.032 (4)	0.002 (4)	0.015 (3)	−0.001 (4)
C43	0.025 (4)	0.023 (6)	0.033 (4)	0.002 (4)	0.011 (3)	0.001 (4)
C44	0.022 (3)	0.026 (4)	0.031 (3)	0.009 (5)	0.006 (3)	−0.007 (5)
C45	0.027 (4)	0.031 (5)	0.038 (4)	0.002 (4)	0.022 (4)	−0.009 (4)
C46	0.030 (5)	0.026 (5)	0.036 (4)	0.001 (4)	0.021 (4)	−0.001 (4)

### (II) Dichlorido[(1S,5S)-4-mesityl-1,2,8,8-tetramethyl-2,4-diazabicyclo[3.2.1]octan-3-ylidene-κC3](triphenylphosphane-κP)palladium(IV) Geometric parameters (Å, º)

**Table:**

Pd1—C1	2.048 (7)	C15—C16	1.381 (11)
Pd1—Cl1	2.309 (2)	C15—H15A	0.9500
Pd1—Cl2	2.311 (2)	C16—C19	1.494 (11)
Pd1—P1	2.355 (2)	C17—H17A	0.9800
P1—C41	1.819 (8)	C17—H17B	0.9800
P1—C31	1.827 (7)	C17—H17C	0.9800
P1—C21	1.832 (8)	C18—H18A	0.9800
N1—C1	1.324 (9)	C18—H18B	0.9800
N1—C2	1.450 (10)	C18—H18C	0.9800
N1—C3	1.526 (8)	C19—H19A	0.9800
N2—C1	1.338 (9)	C19—H19B	0.9800
N2—C11	1.452 (9)	C19—H19C	0.9800
N2—C7	1.507 (8)	C21—C26	1.386 (11)
C2—H2A	0.9800	C21—C22	1.390 (11)
C2—H2B	0.9800	C22—C23	1.390 (12)
C2—H2C	0.9800	C22—H22A	0.9500
C3—C4	1.514 (11)	C23—C24	1.380 (13)
C3—C8	1.536 (14)	C23—H23A	0.9500
C3—C5	1.561 (8)	C24—C25	1.364 (12)
C4—H4A	0.9800	C24—H24A	0.9500
C4—H4B	0.9800	C25—C26	1.387 (11)
C4—H4C	0.9800	C25—H25A	0.9500
C5—C6	1.544 (13)	C26—H26A	0.9500
C5—H5A	0.9900	C31—C32	1.379 (11)
C5—H5B	0.9900	C31—C36	1.409 (11)
C6—C7	1.527 (10)	C32—C33	1.387 (11)
C6—H6A	0.9900	C32—H32A	0.9500
C6—H6B	0.9900	C33—C34	1.386 (14)
C7—C8	1.536 (10)	C33—H33A	0.9500
C7—H7A	1.0000	C34—C35	1.371 (13)
C8—C10	1.530 (10)	C34—H34A	0.9500
C8—C9	1.538 (10)	C35—C36	1.378 (11)
C9—H9A	0.9800	C35—H35A	0.9500
C9—H9B	0.9800	C36—H36A	0.9500
C9—H9C	0.9800	C41—C42	1.402 (11)
C10—H10A	0.9800	C41—C46	1.409 (11)
C10—H10B	0.9800	C42—C43	1.384 (12)
C10—H10C	0.9800	C42—H42A	0.9500
C11—C12	1.402 (10)	C43—C44	1.377 (10)
C11—C16	1.413 (10)	C43—H43A	0.9500
C12—C13	1.397 (11)	C44—C45	1.365 (13)
C12—C17	1.505 (11)	C44—H44A	0.9500
C13—C14	1.387 (12)	C45—C46	1.373 (12)
C13—H13A	0.9500	C45—H45A	0.9500
C14—C15	1.394 (11)	C46—H46A	0.9500
C14—C18	1.493 (12)		
			
C1—Pd1—Cl1	88.7 (2)	C12—C13—H13A	118.1
C1—Pd1—Cl2	88.6 (2)	C13—C14—C15	116.2 (7)
Cl1—Pd1—Cl2	173.53 (9)	C13—C14—C18	122.3 (8)
C1—Pd1—P1	177.6 (2)	C15—C14—C18	121.4 (8)
Cl1—Pd1—P1	93.58 (7)	C16—C15—C14	123.6 (7)
Cl2—Pd1—P1	89.18 (7)	C16—C15—H15A	118.2
C41—P1—C31	101.5 (3)	C14—C15—H15A	118.2
C41—P1—C21	105.7 (4)	C15—C16—C11	117.8 (7)
C31—P1—C21	103.3 (3)	C15—C16—C19	119.7 (7)
C41—P1—Pd1	116.6 (2)	C11—C16—C19	122.4 (7)
C31—P1—Pd1	119.1 (2)	C12—C17—H17A	109.5
C21—P1—Pd1	109.2 (2)	C12—C17—H17B	109.5
C1—N1—C2	120.8 (6)	H17A—C17—H17B	109.5
C1—N1—C3	119.9 (6)	C12—C17—H17C	109.5
C2—N1—C3	119.3 (6)	H17A—C17—H17C	109.5
C1—N2—C11	122.0 (6)	H17B—C17—H17C	109.5
C1—N2—C7	121.6 (6)	C14—C18—H18A	109.5
C11—N2—C7	116.2 (6)	C14—C18—H18B	109.5
N1—C1—N2	119.0 (6)	H18A—C18—H18B	109.5
N1—C1—Pd1	119.8 (5)	C14—C18—H18C	109.5
N2—C1—Pd1	121.2 (5)	H18A—C18—H18C	109.5
N1—C2—H2A	109.5	H18B—C18—H18C	109.5
N1—C2—H2B	109.5	C16—C19—H19A	109.5
H2A—C2—H2B	109.5	C16—C19—H19B	109.5
N1—C2—H2C	109.5	H19A—C19—H19B	109.5
H2A—C2—H2C	109.5	C16—C19—H19C	109.5
H2B—C2—H2C	109.5	H19A—C19—H19C	109.5
C4—C3—N1	111.0 (7)	H19B—C19—H19C	109.5
C4—C3—C8	115.4 (6)	C26—C21—C22	120.0 (7)
N1—C3—C8	105.9 (6)	C26—C21—P1	117.9 (6)
C4—C3—C5	112.7 (7)	C22—C21—P1	122.0 (6)
N1—C3—C5	107.2 (5)	C21—C22—C23	119.6 (9)
C8—C3—C5	104.1 (8)	C21—C22—H22A	120.2
C3—C4—H4A	109.5	C23—C22—H22A	120.2
C3—C4—H4B	109.5	C24—C23—C22	119.9 (8)
H4A—C4—H4B	109.5	C24—C23—H23A	120.0
C3—C4—H4C	109.5	C22—C23—H23A	120.0
H4A—C4—H4C	109.5	C25—C24—C23	120.3 (8)
H4B—C4—H4C	109.5	C25—C24—H24A	119.9
C6—C5—C3	105.8 (8)	C23—C24—H24A	119.9
C6—C5—H5A	110.6	C24—C25—C26	120.8 (8)
C3—C5—H5A	110.6	C24—C25—H25A	119.6
C6—C5—H5B	110.6	C26—C25—H25A	119.6
C3—C5—H5B	110.6	C21—C26—C25	119.3 (8)
H5A—C5—H5B	108.7	C21—C26—H26A	120.3
C7—C6—C5	102.5 (6)	C25—C26—H26A	120.3
C7—C6—H6A	111.3	C32—C31—C36	119.2 (7)
C5—C6—H6A	111.3	C32—C31—P1	124.2 (6)
C7—C6—H6B	111.3	C36—C31—P1	116.6 (6)
C5—C6—H6B	111.3	C31—C32—C33	121.3 (8)
H6A—C6—H6B	109.2	C31—C32—H32A	119.3
N2—C7—C6	108.1 (6)	C33—C32—H32A	119.3
N2—C7—C8	109.1 (6)	C34—C33—C32	119.1 (8)
C6—C7—C8	103.7 (6)	C34—C33—H33A	120.4
N2—C7—H7A	111.8	C32—C33—H33A	120.4
C6—C7—H7A	111.8	C35—C34—C33	119.8 (8)
C8—C7—H7A	111.8	C35—C34—H34A	120.1
C10—C8—C7	115.6 (6)	C33—C34—H34A	120.1
C10—C8—C3	113.9 (6)	C34—C35—C36	121.8 (8)
C7—C8—C3	98.1 (6)	C34—C35—H35A	119.1
C10—C8—C9	107.8 (6)	C36—C35—H35A	119.1
C7—C8—C9	109.3 (6)	C35—C36—C31	118.7 (8)
C3—C8—C9	111.9 (6)	C35—C36—H36A	120.6
C8—C9—H9A	109.5	C31—C36—H36A	120.6
C8—C9—H9B	109.5	C42—C41—C46	117.1 (8)
H9A—C9—H9B	109.5	C42—C41—P1	118.9 (6)
C8—C9—H9C	109.5	C46—C41—P1	124.0 (6)
H9A—C9—H9C	109.5	C43—C42—C41	121.3 (7)
H9B—C9—H9C	109.5	C43—C42—H42A	119.3
C8—C10—H10A	109.5	C41—C42—H42A	119.3
C8—C10—H10B	109.5	C44—C43—C42	119.4 (9)
H10A—C10—H10B	109.5	C44—C43—H43A	120.3
C8—C10—H10C	109.5	C42—C43—H43A	120.3
H10A—C10—H10C	109.5	C45—C44—C43	120.7 (10)
H10B—C10—H10C	109.5	C45—C44—H44A	119.6
C12—C11—C16	121.0 (7)	C43—C44—H44A	119.6
C12—C11—N2	120.5 (7)	C44—C45—C46	120.5 (8)
C16—C11—N2	118.3 (6)	C44—C45—H45A	119.8
C13—C12—C11	117.4 (7)	C46—C45—H45A	119.8
C13—C12—C17	117.2 (7)	C45—C46—C41	120.9 (8)
C11—C12—C17	125.3 (7)	C45—C46—H46A	119.5
C14—C13—C12	123.8 (8)	C41—C46—H46A	119.5
C14—C13—H13A	118.1		
			
C2—N1—C1—N2	−179.2 (7)	C13—C14—C15—C16	1.1 (11)
C3—N1—C1—N2	2.9 (9)	C18—C14—C15—C16	179.6 (7)
C2—N1—C1—Pd1	0.8 (9)	C14—C15—C16—C11	0.9 (11)
C3—N1—C1—Pd1	−177.1 (5)	C14—C15—C16—C19	−178.4 (7)
C11—N2—C1—N1	−169.9 (6)	C12—C11—C16—C15	−3.1 (10)
C7—N2—C1—N1	5.3 (10)	N2—C11—C16—C15	−178.2 (6)
C11—N2—C1—Pd1	10.1 (9)	C12—C11—C16—C19	176.1 (7)
C7—N2—C1—Pd1	−174.7 (5)	N2—C11—C16—C19	1.0 (10)
C1—N1—C3—C4	−170.2 (7)	C41—P1—C21—C26	154.5 (6)
C2—N1—C3—C4	11.8 (10)	C31—P1—C21—C26	−99.3 (6)
C1—N1—C3—C8	−44.2 (8)	Pd1—P1—C21—C26	28.4 (7)
C2—N1—C3—C8	137.8 (7)	C41—P1—C21—C22	−27.4 (8)
C1—N1—C3—C5	66.4 (10)	C31—P1—C21—C22	78.8 (7)
C2—N1—C3—C5	−111.6 (9)	Pd1—P1—C21—C22	−153.5 (6)
C4—C3—C5—C6	145.1 (7)	C26—C21—C22—C23	1.2 (13)
N1—C3—C5—C6	−92.5 (8)	P1—C21—C22—C23	−176.9 (7)
C8—C3—C5—C6	19.4 (6)	C21—C22—C23—C24	−1.0 (14)
C3—C5—C6—C7	11.8 (7)	C22—C23—C24—C25	1.1 (15)
C1—N2—C7—C6	−83.3 (8)	C23—C24—C25—C26	−1.5 (14)
C11—N2—C7—C6	92.2 (7)	C22—C21—C26—C25	−1.5 (12)
C1—N2—C7—C8	28.9 (9)	P1—C21—C26—C25	176.6 (6)
C11—N2—C7—C8	−155.6 (6)	C24—C25—C26—C21	1.7 (13)
C5—C6—C7—N2	76.6 (7)	C41—P1—C31—C32	115.9 (7)
C5—C6—C7—C8	−39.2 (7)	C21—P1—C31—C32	6.5 (8)
N2—C7—C8—C10	57.3 (9)	Pd1—P1—C31—C32	−114.7 (6)
C6—C7—C8—C10	172.4 (6)	C41—P1—C31—C36	−61.3 (7)
N2—C7—C8—C3	−64.2 (6)	C21—P1—C31—C36	−170.7 (6)
C6—C7—C8—C3	50.8 (6)	Pd1—P1—C31—C36	68.1 (7)
N2—C7—C8—C9	179.1 (6)	C36—C31—C32—C33	0.7 (12)
C6—C7—C8—C9	−65.9 (8)	P1—C31—C32—C33	−176.4 (7)
C4—C3—C8—C10	71.2 (8)	C31—C32—C33—C34	0.3 (13)
N1—C3—C8—C10	−51.9 (7)	C32—C33—C34—C35	−0.4 (14)
C5—C3—C8—C10	−164.8 (6)	C33—C34—C35—C36	−0.6 (15)
C4—C3—C8—C7	−166.0 (5)	C34—C35—C36—C31	1.6 (14)
N1—C3—C8—C7	70.8 (6)	C32—C31—C36—C35	−1.6 (12)
C5—C3—C8—C7	−42.1 (6)	P1—C31—C36—C35	175.7 (7)
C4—C3—C8—C9	−51.3 (8)	C31—P1—C41—C42	160.9 (6)
N1—C3—C8—C9	−174.5 (6)	C21—P1—C41—C42	−91.5 (6)
C5—C3—C8—C9	72.7 (7)	Pd1—P1—C41—C42	30.0 (7)
C1—N2—C11—C12	75.1 (8)	C31—P1—C41—C46	−19.7 (7)
C7—N2—C11—C12	−100.3 (8)	C21—P1—C41—C46	87.8 (7)
C1—N2—C11—C16	−109.7 (8)	Pd1—P1—C41—C46	−150.7 (6)
C7—N2—C11—C16	74.8 (8)	C46—C41—C42—C43	−0.5 (11)
C16—C11—C12—C13	3.4 (10)	P1—C41—C42—C43	178.9 (6)
N2—C11—C12—C13	178.4 (6)	C41—C42—C43—C44	1.5 (12)
C16—C11—C12—C17	−172.3 (7)	C42—C43—C44—C45	−1.3 (12)
N2—C11—C12—C17	2.7 (11)	C43—C44—C45—C46	0.2 (13)
C11—C12—C13—C14	−1.4 (11)	C44—C45—C46—C41	0.8 (13)
C17—C12—C13—C14	174.6 (7)	C42—C41—C46—C45	−0.6 (12)
C12—C13—C14—C15	−0.8 (11)	P1—C41—C46—C45	−179.9 (7)
C12—C13—C14—C18	−179.3 (7)		

### (II) Dichlorido[(1S,5S)-4-mesityl-1,2,8,8-tetramethyl-2,4-diazabicyclo[3.2.1]octan-3-ylidene-κC3](triphenylphosphane-κP)palladium(IV) Hydrogen-bond geometry (Å, º)

**Table:**

*D*—H···*A*	*D*—H	H···*A*	*D*···*A*	*D*—H···*A*
C42—H42*A*···Cl2	0.95	2.62	3.447 (8)	146
C15—H15*A*···Cl2^i^	0.95	2.71	3.535 (8)	146

Symmetry code: (i) *x*, *y*+1, *z*.
